# Possible Ameliorative Effects of the Royal Jelly on Hepatotoxicity and Oxidative Stress Induced by Molybdenum Nanoparticles and/or Cadmium Chloride in Male Rats

**DOI:** 10.3390/biology11030450

**Published:** 2022-03-16

**Authors:** Reham Z. Hamza, Rasha A. Al-Eisa, Nahla S. El-Shenawy

**Affiliations:** 1 Zoology Department, Faculty of Science, Zagazig University, Zagazig 44519, Egypt; 2 Biology Department, Main Campus, College of Science, Taif University, Taif 21944, Saudi Arabia; stardust20117@gmail.com; 3 Zoology Department, Faculty of Science, Suez Canal University, Ismailia 41522, Egypt; elshenawy_nahla@hotmail.com or

**Keywords:** biochemical, lipid profile, enzyme activity, caspase-3, electron micrograph

## Abstract

**Simple Summary:**

The aim of the present investigation is valuable due to the importance of possible contaminations and negative effects of these cadmium chloride and molybdenum nanoparticles. The physicochemical properties of molybdenum nanoparticles have been characterized, as well as their ultrastructural organization. A rat experimental model was then employed to assess the liver toxicity of molybdenum nanoparticles, even in combination with CdCl_2_. The toxicity of molybdenum nanoparticles and cadmium chloride was estimated via liver damage by means of chemico-biological markers of liver injury, serum lipids, inflammation, oxidative status, and histological and immunohistochemistry patterns. Moreover, the possible effects of royal jelly were evaluated. The results clarified that both chemicals induced hepatic toxicity with the excessive triggering of reactive oxygen species that induced severe oxidative injury, histological alterations in the hepatic structure, and hepatic ultrastructure. These results are concurrent with obtaining normal biochemical levels in groups either treated with royal jelly or even a combination of royal jelly and two xenobiotics. The royal jelly is considered an essential potential source of natural antioxidants capable of frustrating the effects of oxidative injury, which is considered the main cause of many diseases.

**Abstract:**

The present study aimed to investigate the effect of the royal jelly (RJ) on hepatotoxicity induced by molybdenum nanoparticles (MoO_3_-NPs), cadmium chloride (CdCl_2_), or their combination in male rats at biochemical, inflammation, immune response, histological, and ultrastructural levels. The physicochemical properties of MoO_3_-NPs have been characterized, as well as their ultrastructural organization. A rat experimental model was employed to assess the liver toxicity of MoO_3_-NPs, even in combination with CdCl_2_. Different cellular studies indicate divergent mechanisms, from increased reactive oxygen species production to antioxidative damage and cytoprotective activity. Seventy male rats were allocated to groups: (i) control; (ii) MoO_3_-NPs (500 mg/kg); (iii) CdCl_2_ (6.5 mg/kg); (iv) RJ (85 mg/kg diluted in saline); (v) MoO_3_-NPs followed by RJ (30 min after the MoO_3_-NPs dose); (vi) CdCl_2_ followed by RJ; and (vii) a combination of MoO_3_-NPs and CdCl_2_, followed by RJ, for a total of 30 successive days. Hepatic functions, lipid profile, inflammation marker (CRP), antioxidant biomarkers (SOD, CAT, GPx, and MDA), and genotoxicity were examined. Histological changes, an immunological marker for caspase-3, and transmission electron microscope variations in the liver were also investigated to indicate liver status. The results showed that RJ alleviated the hepatotoxicity of MoO_3_-NPs and/or CdCl_2_ by improving all hepatic vitality markers. In conclusion, the RJ was more potent and effective as an antioxidant over the oxidative damage induced by the combination of MoO_3_-NPs and CdCl_2_.

## 1. Introduction

Pollution increases due to rapid growth of environmental problems. Molybdenum (Mo) is a transition metal that belongs to the periodic table’s group VI-B. Mo is regarded as a vital trace element for plants, animals, and microbes because of its critical functions in various molybdoenzymes, where molybdenum serves as a cofactor. Xanthine oxidase, aldehyde oxidase, and sulfite oxidase are examples of molybdoenzymes essential for biometabolic functions in mammals [1,2].

Molybdenum oxides and molybdenum nanoparticles (Mo-NPs) have been used in many fields; Mo-NPs are used as a lubricant, smoke suppresser, and fire retardant [3]. Heavy metals ions, such as cadmium chloride (CdCl_2_), are toxic chemicals released into the soil, water, and air, which then enter the food chain [4], and thus, human beings are exposed to Mo-NPs and CdCl_2_ in different ways that make the study of these pollutants inducing toxicity necessary.

Molybdenum has the potential to be harmful to people. On the other hand, its poisoning mechanism/s is mostly unknown. Excessive consumption of molybdenum-rich grains, seeds, and legumes has been linked to Mo deposits in soft tissues and joints, which can lead to arthritic symptoms. Furthermore, molybdenum overdose has been linked to severe gastrointestinal discomfort, including diarrhea, ruminant coma, and death from heart failure [5].

The nanotechnology sector has developed at an exponential rate in recent years, and this trend is expected to continue in the foreseeable future [6,7,8]. Attia [9] found that copper nanoparticles induced oxidative liver damage. Regrettably, our tremendous advancement in nanotechnology has outpaced studies into the effects of nanoparticles on human well-being. Nanoscale materials function in biological systems via methods and reactions that are quite different from their bulk or macro counterparts. Quantum effects prevail in the world of the very small, and their implications for the living environment are only just beginning to be understood.

Molybdenum nanoparticles (Mo-NPs) have a wide range of uses in the electron industry. Excess Mo-NPs in industrial effluents has been linked to negative health consequences in animals and humans [5,6].

There is less evidence on the hazardous and innocuous effects of Mo-NPs. In previous studies [6,10], Mo-NPs have been shown to cause cytotoxicity and oxidative stress in mice skin fibroblast cells, as well as cytoprotective effects in breast cancer cells and the fibrosarcoma cell line. Therefore, it is critical to be aware of the possible dangers of molybdenum oxide nanoparticles (MoO_3_-NPs).

Cadmium (Cd) is usually found in combination with other metals. Smelting and refining of Zn, Pb, and Cu ores produces the majority of the world’s Cd. Cd is mostly utilized in industry for electroplating and Cd-bearing alloys. Air, drinking water, and food can all be contaminated with Cd. Industrial waste, pesticides, and fertilizers, galvanized pipes carrying drinking water, cigarette smoke, coal, oil, and wood use, and the smelting of rubber types are all sources of Cd pollution in the air and water [11].

RJ is a creamy fluid released by worker bees’ mandibular and hypopharyngeal glands that is the food that all honeybee larvae eat for the first three days after they are born. The worker bee larvae then begin to ingest worker jelly, which largely consists of honey and pollen. The RJ is eaten by queen bee larvae [12,13].

RJ, which is produced by the incomplete digestion of honeydew in the stomach of worker bees, is essential for the development of the queen bee [14]. RJ is a blend of glucose, lipid, protein, minerals, vitamins [15], phosphorous compounds, gel, nucleic acids, and acetylcholine, which are crucial for the nutritional properties of RJ [16].

As a bee product, RJ is utilized to preserve human health [17]. In addition, royalactin is a unique component in RJ that causes bee larvae to differentiate into queens. This jelly is a type of functional food and dietary supplement with various biological actions, including anti-tumor, anti-allergy, anti-inflammatory, and immunological modulation [17]. These functions are linked to RJ’s many functional ingredients, including proteins, lipids, carbs, vitamins, and minerals [17]. RJ proteins (MRJP1-MRJP9) are water-soluble proteins with a wide range of physiological functions. They have antiviral, anticancer, and anti-hepatic damage properties, according to Habashy and Abu-Serie [18]. Moreover, RJ has significant nephroprotective benefits in male Wister albino rats intoxicated with CCl_4_ by avoiding oxidative stress (ROS scavenger) and improving the former biochemical parameters [19].

Because of its critical involvement in the metabolism, transport, and clearance of foreign chemicals, the liver plays a critical role in determining drug toxicity. Cytochrome P450 enzymes (CYP450) metabolize most of the xenobiotic compounds in the liver, with three CYP families accounting for 77% of documented xenobiotic biotransformation [20]. However, many parameters can impact the liver’s metabolic function, and drug-metabolizing enzyme inhibition and induction are common causes of drug–drug interactions [21]. Therefore, the present study objective was to investigate the effect of the RJ on the hepatotoxicity induced by MoO_3_-NPs, CdCl_2,_ or their combination in male rats using chemico-biological markers of liver injury, serum lipids, inflammation, oxidative status, and histological and ultrastructural patterns.

## 2. Materials and Methods

### 2.1. Chemicals

Pure royal jelly capsules were purchased from Pharco pharmaceuticals Co. Cairo, Egypt. Whereas CdCl_2_ and ammonium molybdate were provided by Sigma-Aldrich, St. Louis, MO, USA.

### 2.2. Synthesis of MoO_3_-NPs and Their Characterization

#### 2.2.1. MoO_3_-NPs Synthesis

The syntheses of MoO_3_-NPs were carried out by using ammonium molybdate tetrahydrate (NH_4_)6Mo_7_O_24_·4H_2_O as the main source of molybdenum in the experiment, where (NH4)_6_Mo_7_O_24_·4H_2_O (1 mmol) was dissolved in 40 mL of distilled water, while 20 mmol of KNO_3_ and NaNO_3_, Ca(NO_3_)_2_ (5 mmol), and La (NO_3_)_3_ (3 mmol) were dissolved in 30 mL of distilled water. Then, under continuous magnetic stirring for 4 h and by the slow addition of (NH_4_) 6Mo_7_O_24_ of NO_3_ solution, form a homogeneous aqueous solution. The pH value ranges between 1 and 4. It is controlled by using an HNO_3_ solution. Then the formed solution was transferred into an autoclave and heated to 200 °C for 1 day using a controlled oven, followed by cooling at room temperature. Filtration yields products with a dark white-yellow color, which can be washed several times with deionized water and dried in a vacuum at 60 °C. The product, MoO_3_-NPs, was annealed at 600 °C in oxygen for 3 h.

#### 2.2.2. MoO_3_-NPs Physicochemical Characterization Methods 

With Cu KR radiation (0.15406 nm), the X-ray diffraction (XRD) spectra were recorded on a D/MAX-2500 diffraction meter. The operation voltage was maintained at 40 kV and 200 mA. 

The transmission electron microscope images (TEM) were taken with a JEOL-JEM2010 operating at 200 kV (JEOL, Tokyo, Japan). Dropping a diluted dispersion of MoO_3_-NPs over a conventional carbon-coated (20–30 nm) sheet on a copper grid produced TEM samples.

Scanning electron microscopy (SEM; JEOL, Tokyo, Japan) was used for obtaining surface images equipped with energy-dispersive X-ray fluorescence analysis (EDX) that was used to investigate the morphology of samples. The product was applied over the silicon substrate for SEM inspection.

Fourier transformation infrared spectroscopy (FT-IR) was acquired with an FT-IR spectrometer. The FT-IR spectra of MoO_3_-NPs were studied on pellets made by combining the nanoparticles with KBr at around a 1:50 volume ratio.

The UV-visible absorption spectroscopic measurements were performed on a HITACHI U-4100 spectrophotometer using a quartz cell with a width of 1 cm. 

Zeta potential measurements is a technique for the assessment of the particle’s surface charge in the solution; this technique is directly related to the stability of MoO_3_-NPs suspension. 

### 2.3. Determination of Half Lethal Dose of MoO_3_-NPs

Male rats were orally delivered a single dose of MoO_3_-NPs suspension at dosage levels 100, 500, or 1000 mg/kg and then examined for symptoms of toxicity and mortality for 15 days to determine the half-lethal dose of MoO_3_-NPs. Because male rats showed no evidence of toxicity or death after 15 days, the LD_50_ of MoO_3_-NPs is predicted to be >1000 mg/kg thus we used 500 mg/kg of MoO_3_-NPs, as recommended by Tice et al. [22], for compounds that showed no signs of mortality even at high dosages.

### 2.4. Animals Groups, Ethics, and Treatment

The animals were cared for in accordance with the Deanship of Scientific Research at Taif University’s inquiry and ethics guidelines for laboratory animal care (approval No. 42-0073).

The experiment was carried out at the Biology department, Taif University. Male rats weighing between 180 and 200 g were obtained and kept at the Animal House of the Faculty of Science. During the experiment period, the rats were placed under typical laboratory conditions (22 °C, 50% relative humidity, and a natural daylight cycle 12/12 h). They were fed with a commercially available pellet diet. Food and drink were freely accessible.

**Figure 1:**
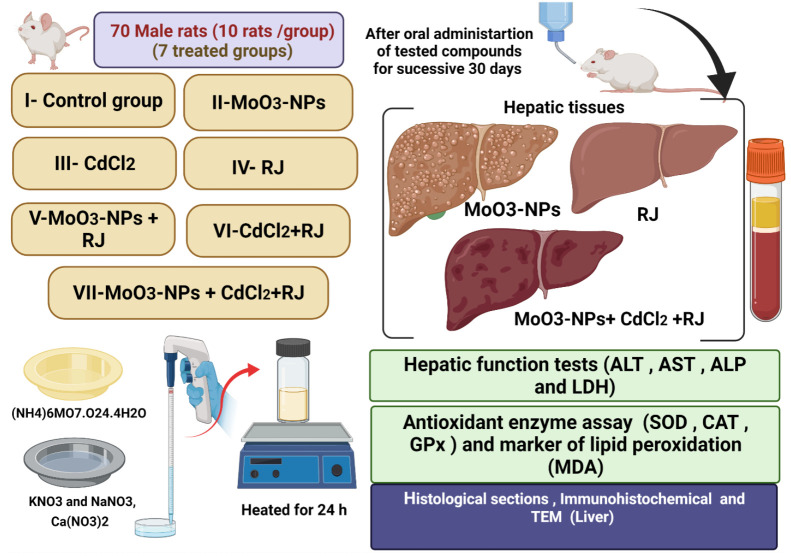
Schematic representation of the experimental design.

The experiment was performed on 70 male albino rats, divided into seven groups, who were administrated the compounds orally (by using a rat oral feeding tube) for 30 successive days:
Group I served as the control; the animals received 0.9% NaCl (physiological saline). Group II was given 500 mg/kg of MoO_3_-NPs dispersed in saline.Group III was administered with CdCl_2_ at a dose of 5 mg/kg dissolved in physiological saline [23].Group IV was given royal jelly (RJ; 85 mg/kg diluted in saline; this dose is equivalent to 250 mg crude RJ) [24].Group V received MoO_3_-NPs as the first dosage, followed by RJ 30 min after the MoO_3_-NPs were administered.Group VI was given CdCl_2_ first, followed by RJ, as previously described.Group VII was treated with a combination of MoO_3_-NPs and CdCl_2_, followed by RJ treatment at the same dosages (Figure 1). All animals were treated orally for 30 consecutive days.

### 2.5. Sample Collection 

To separate the serum, blood samples were taken and centrifuged at 3000 rpm for 10 min at 4 °C. The obtained serum was kept at 20 °C for further testing. The rats were quickly decapitated after mild anesthesia with xylene/ketamine HCl (75 mg/kg/10 mg/kg intraperitoneally), and the liver was removed, weighed, and homogenized (Figure 1). After centrifuging homogenates at 4500 *g* for 20 min at 4 °C, the supernatants were collected and kept at 20 °C for future antioxidant enzyme investigation.

### 2.6. Biochemical Investigation

#### 2.6.1. Liver Function Assessment and Inflammation Markers

On the 30th day of treatment, the heparinized non-hemolyzed serum lactate dehydrogenase (LDH) activity of 12 h post-fasted rats was assessed. As previously reported, serum LDH activity was determined using a Spectrum Diagnostics LDH reagent/assay kit (MDSS GmbH Schigraben, Hannover, Germany) according to the manufacturer’s instructions. Further, the activity of alanine aminotransferase (ALT), aspartate aminotransferase (AST), and alkaline phosphatase (ALP) were evaluated in the serum using commercial kits (Spinreact, Girona, Spain) according to the manufacturer’s instructions.

The lipid profile was evaluated by the determination of serum total cholesterol (TC) and triglycerides (TG), according to Carr et al. [25] and Warnick et al. [26], respectively. Low-density lipoprotein cholesterol (LDL-c) and very-low-density lipoprotein cholesterol (vLDL-c) were calculated using the Friedwald et al. [27] and Norbert [28] formulae, respectively: vLDL-c (mg/dL) = TG/5 + HDL-c, and LDL-c (mg/dL) = TC − (TG/5 + HDL-c).

The sandwich enzyme-linked immunosorbent assay (ELISA) technique was conducted according to the manufacturer’s (Ebio-Science) instructions (Bio-Tek Instruments, Inc.) to determine interleukin 6 (IL-6) (Cat. No. BMS625), tumor necrosis factor-alpha (TNF-α) (Cat. No. BMS622), and C-reactive protein (CRP). 

#### 2.6.2. Assessment of Antioxidant and Oxidative Stress Indices

A small piece of the liver (0.25 g) was separated and homogenized using cold Tris–HCl buffer (pH 7.4) and was centrifuged to obtain the supernatant that was used for further biochemical assays. According to Ohkawa et al. [29], malondialdehyde (MDA) was detected as a lipid peroxidation (LPO) product using thiobarbituric acid-reactive compounds.

According to Sun et al. [30], superoxide dismutase (SOD) activity was assessed. The degree of inhibition of this process is then used to calculate SOD activity. Aebi [31] published the technique for determining CAT activity, which involves measuring the rate of breakdown of H_2_O_2_ at 240 nm (Spectrophotometer SP-2200, Bioespectro, Curitiba, Brazil). The results were reported in U/g protein. Glutathione peroxidase (GPx) activity was assessed [32]. 

### 2.7. Histological, Apoptosis, and Immunohistochemically Assessment

Fixed samples were processed following a conventional procedure for hematoxylin/eosin staining of liver tissue sections. A light microscope was used to view the stained slides, and photomicrographs of the tissue samples were taken.

The obtained liver slices (4-m thickness) were blocked with 0.1 percent hydrogen peroxide-containing methanol for 15 min to disrupt the endogenous peroxidase and study apoptosis-related proteins. After blocking, the sections were treated overnight at 4 °C with a rabbit polyclonal caspase-3 antibody. The initial magnification of the images was 100 (Nikon, Tokyo, Japan).

The color intensity of the protein in the immunohistochemical sections was assessed in a semi-quantitative manner. + (weak immunoreactivity), ++ (moderate immunoreactivity), +++ (strong immunoreactivity), and ++++ (very strong immunoreactivity) were used to categorize the intensity.

### 2.8. Statistical and Data Analysis

The data were expressed as mean ± SE. For data comparison comparing several groups, a One-Way Analysis of Variance was employed, using a least significant difference post-hoc test. Statistical significance was defined as a value of *p* ≤ 0.05.

## 3. Results

Establishing new nanoparticles and the characterization of existing ones are critical in nanotoxicology research to provide us with a real picture of how to mimic the very tiny particles or any type of pollutants at a very minute size that resembles residues to enable us to understand the nature of the toxicity of these very small particles in the nanoscale and their deleterious effect on public health. The present study was conducted to evaluate the adverse effects of both MoO_3_-NPs and/or CdCl_2_ alone and in combination with RJ on liver markers. The antioxidant enzymes, such as SOD, CAT, and GPx, as well as the liver tissues, were used to examine histopathological, immunological, and TEM variations.

### 3.1. The Infrared Spectrum of Synthesized MoO_3_-NPs

**Figure 2:**
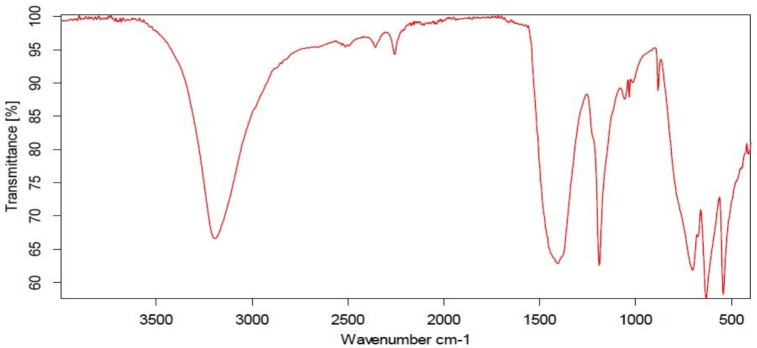
IR of MoO_3_-NPs.

The sample’s FT-IR spectra are measured in the region of 4000–400/cm (Figure 2). The fundamental vibrational modes of Mo=O are responsible for the distinctive bands found at 495, 600, and 990/cm. In general, MoO_3_ has 17 active IR modes.

### 3.2. UV-Vis Spectrum of Synthesized MoO_3_-NPs

**Figure 3:**
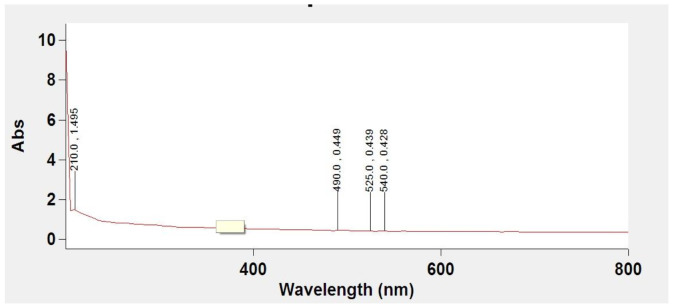
The absorption spectra of prepared MoO_3_-NPs particles.

The UV–Vis absorption spectra of synthesized MoO_3_ show that the absorption spectra curves display a broad absorption in the range of 200–600 nm wavelength (Figure 3). 

### 3.3. Scanning Electron Microscope (SEM) 

**Figure 4:**
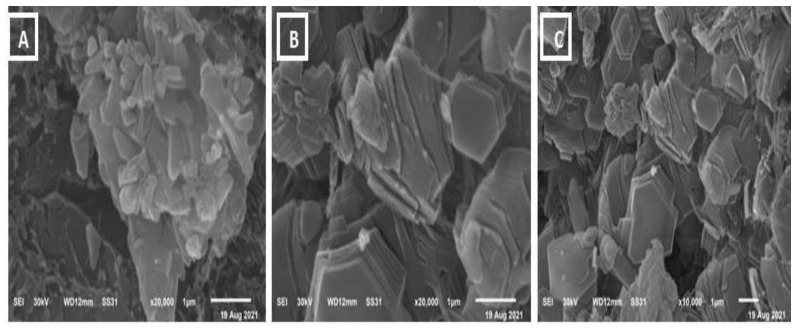
SEM of Ammonium Molybdate (**A**–**C**).

**Figure 5:**
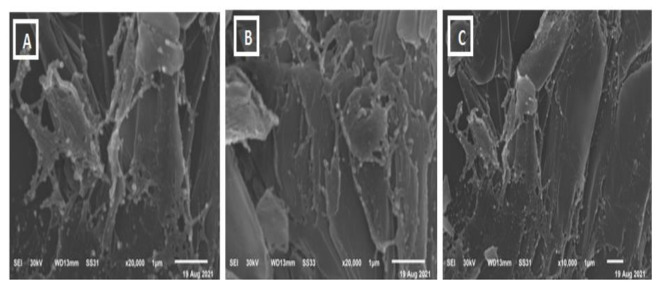
SEM MoO_3_-NPs (**A**–**C**).

**Figure 6:**
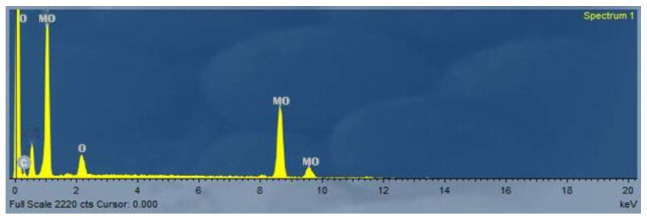
EDX of MoO_3_-NPs.

The morphological studies of the sample are observed from SEM images. SEM analysis is an important characterization technique for topographic studies of the sample and gives information regarding the growth mechanism, shape, and size of the particles. Figure 4A–C shows the images of samples A (ammonium molybdate) taken at different magnifications. The prepared samples contain rod and sphere-like mixed structures with irregular shapes and dimensions. Meanwhile, SEM analysis of the MoO_3_-NPs sample revealed thin filaments and small dots (Figure 5A–C). Chemical composition analysis by EDX confirms the presence of only Mo, O, and traces of C, which confirms the high purity of the material (Figure 6) since no other metals were detected.

### 3.4. Transmission Electron Micrographs (TEM) of Ammonium Molybdate and MoO_3_-NPs

**Figure 7:**
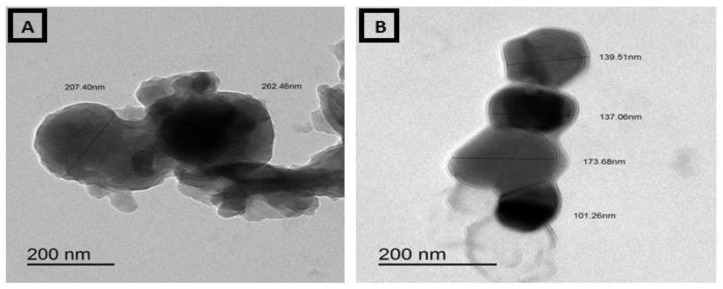
TEM of ammonium molybdate (**A**,**B**).

**Figure 8:**
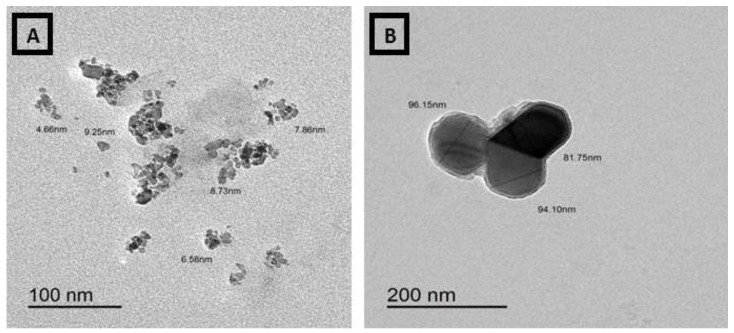
TEM of MoO_3_-NPs (**A**,**B**).

The morphology of ammonium molybdate appeared as a gelatinous, spherical, and filamentous mixed form. Particle sizes ranged from 137.68 to 262.46 nm (Figure 7). Meanwhile, the MoO_3_ sample was determined by the TEM system. Figure 8 shows a typical TEM image of the MoO_3_. The particle size and uniform morphological properties of the MoO_3_ are determined using the TEM micrographs. The MoO_3_-NPs were found within the range of 81–96 nm with medium spherical granules in a sphere-like shape (Figure 8).

### 3.5. Zeta Deviation (6.49 mV)

**Figure 9:**
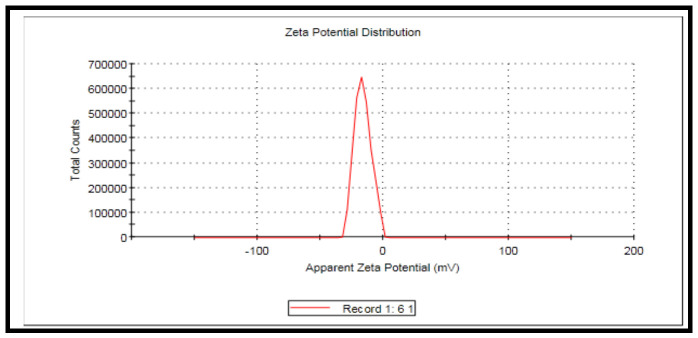
Showing the Zeta potential curve of the prepared MoO_3_-NPs (−16.2 mV).

The diameter of (MoO_3_-NPs) and Zeta potential are presented in Figure 9. The Zeta potential was −16.2 mV. The Zeta potential and size of (MoO_3_-NPs) were determined, and tge Zeta potential decreases while the size of particles enlarge by amplifying the (MoO_3_-NPs) surface area and increasing the stability of the synthesized nanoparticles. The Zeta potential values revealed the stability of MoO_3_-NPs nanoparticles. The pH effect on the size of particles and the Zeta potential distributions of MoO_3_-NPs were evaluated by drop-wise addition of 0.1 M HCl.

### 3.6. Biochemical Evaluation

Table 1 shows the serum enzyme activity results of all groups. The AST, ALT, ALP, and LDH activities were increased in the MoO_3_-NPs and CdCl_2_ groups compared to the control. The data indicated that cell membrane disruption occurred. The biuret reaction is the most extensively used technique for quantifying serum protein. This process is based on serum proteins reacting with copper sulfate in sodium hydroxide to generate a violet biuret complex. The violet color intensity was measured using a DRE 3000 HACH spectrophotometer and is proportional to the protein content.

The total protein level decreased in MoO_3_-NPs and CdCl_2_-treated rats compared to control. The 85 mg/kg of RJ was determined to be nontoxic for the enzyme activity. A decrease in the enzyme activity was observed when the rats were treated with RJ after MoO_3_-NPs or CdCl_2_ was administered. Treatments with RJ elevated the protein content in the MoO_3_-NPs group either as a single treatment or in combination with CdCl_2_.

All the lipid profiles increased with the effect of MoO_3_-NPs or CdCl_2_ except for HDL-c levels (decreased) (Table 2). There was a significant decrease in serum TG in groups treated with MoO_3_-NPs and RJ compared to MoO_3_-NPs alone. The administration of CdCl_2_ resulted in liver dysfunction caused by increasing the levels of TG, TC, LDL-c, and vLDL-c. On the other hand, HDL-c levels declined, and the leakage of enzymes from hepatocytes increased, indicative of cellular damage. The group treated with CdCl_2_ and RJ was able to restore all the previous parameters. In addition, RJ protected the liver cells in the group treated with MoO_3_-NPs and CdCl_2_.

CRP, IL-6, and TNF-α levels were significantly higher in the MoO_3_-NPs and CdCl_2_ groups concerning the control animal (Table 3). The levels of CRP, IL-6, and TNF-α were significantly lower in all RJ groups compared to the high-inflammatory groups treated with MoO_3_-NPs or CdCl_2_ (Table 3), causing liver damage. As a result of an increase in pro-inflammatory markers, they may have a role in the pathogenesis of various liver illnesses. In the current investigation, CRP, TNF-α, and IL-6 levels were notably higher in MoO_3_-NPs or CdCl_2_ treated rats than in control.

Table 4 shows the hepatic malondialdehyde (MDA) level as well as catalase (CAT), superoxide dismutase (SOD), and glutathione peroxidase (GPx) activities. The MoO_3_-NPs group had considerably greater MDA levels in liver tissue than the control group but significantly reduced CAT, SOD, and GPx activities. 

Our results indicated that MDA levels in the hepatic tissues were markedly elevated, and antioxidant enzymes levels were markedly declined in response to treatment with CdCl_2_. The results also showed that MoO_3_-NPs induced oxidative stress in the hepatic tissues of male rats either alone or combined with CdCl_2_, which is an indicator of an increment in the level of lipid peroxidation (LPO) and MDA level, and a decrement in the antioxidant enzyme levels of CAT or GPx and/or SOD.

**Figure 10:**
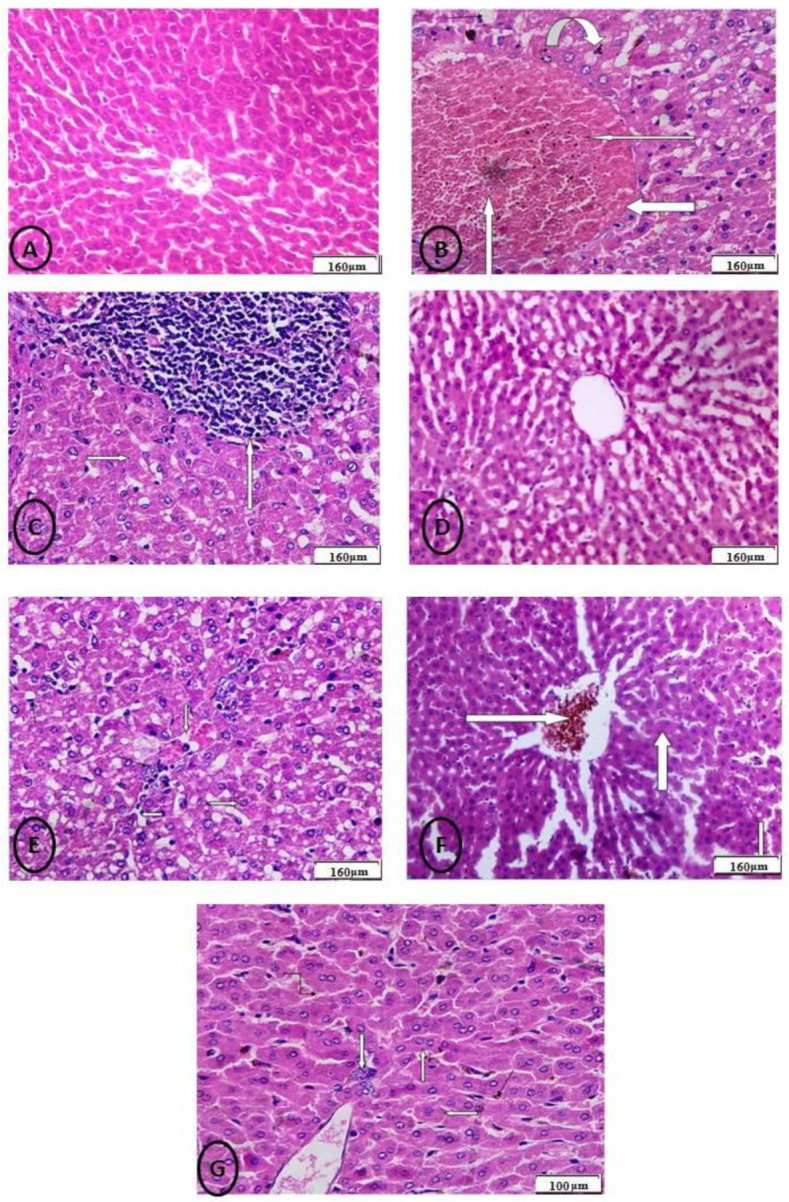
(**A**) Control group: photomicrograph of the cross-section of the hepatic tissues showing normal hepatic structure (160 μm). (**B**): Photomicrograph of a cross-section of the rat liver after administration of MoO_3_-NPs showing severe toxicity in the form of severe damage of liver tissue and severe loss of normal hepatic lobular architecture with and replacement by large areas of hemorrhage and necrosis in the central vein (white thin arrow) surrounded by perivenular fibrosis (inverted white arrow) with microvesicular steatosis in some hepatocytes and accumulation of some bilirubin particles (white thick arrow) indicating obstruction (160 μm). (**C**) Photomicrograph of a cross-section of experimental rat liver treated with CdCl_2_ showing severe loss of normal hepatic lobular architecture, focal necrosis in some hepatocytes, and granulomatous reaction in the form of mononuclear inflammatory cells (granulomatous hepatitis) (white forwarded arrow) (160 μm). (**D**) Photomicrograph of a cross-section of the hepatic tissues of the RJ treated group showing normal hepatic structure (160 μm). (**E**) Photomicrograph of a cross-section of the experimental rat liver after administration of MoO_3_-NPs and RJ showing toxicity in the form of fatty change (ballooning degeneration of hepatocytes) with clear cytoplasm (white arrow), hydropic degeneration in some hepatocytes with vesicular nuclei (white arrow), the portal tract shows a congested portal vein with hemorrhage inside it (white arrow), ductular reaction at the periphery of the portal tract, and infiltration of blood sinusoids by mononuclear inflammatory cells (160 μm). (**F**) Photomicrograph of a cross-section of the experimental rat liver after administration of CdCl_2_ and RJ showing moderate toxicity in the form of hypertrophy of hepatocytes with the appearance of binucleated hepatocytes and increased eosinophilia (thin white arrow), focal necrosis in some hepatocytes (thick white arrow), the central vein shows marked dilatation filled with hemorrhage (160 μm). (**G**) Photomicrograph of a cross-section of hepatic tissues of experimental rat liver treated with the combination of MoO_3_-NPs and CdCl_2_ showing almost normal central vein lined by endothelial cells and hepatic cords radiating from the central vein, the cords are separated by blood sinusoids, which are lined by flat endothelial cells (100 μm).

After the administration of MoO_3_-NPs and RJ, rat liver showed toxicity in the form of fatty change (ballooning degeneration of hepatocytes) with clear cytoplasm, hydropic degeneration in some hepatocytes with vesicular nuclei, and the portal tract showed congested portal vein with a hemorrhage inside it, ductular reaction at the periphery of the portal tract, and infiltration of blood sinusoids by mononuclear inflammatory cells (Figure 10E). The liver, after the administration of CdCl_2_ and RJ, showed moderate toxicity in the form of hypertrophy of hepatocytes with the appearance of binucleated hepatocytes and increased eosinophilia, focal necrosis in some hepatocytes, and the central vein showed marked dilatation filled with hemorrhage (Figure 10F). The rats treated with the combination of MoO_3_-NPs and CdCl_2_ followed by RJ showed an almost normal central vein lined by endothelial cells and hepatic cords radiating from the central vein. Blood sinusoids, which are bordered by flat endothelial cells, separate the cords (Figure 10G).

**Figure 11:**
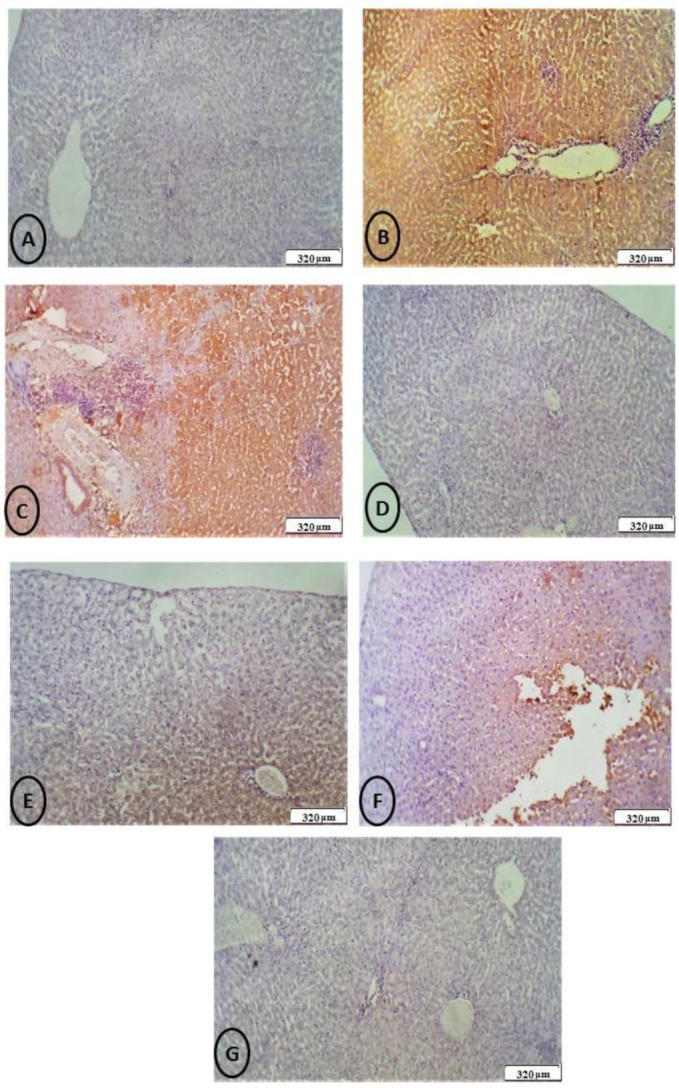
(**A**) Control group: photomicrograph of a cross-section of the hepatic tissues of the control group showing (-Ve) negative caspase-3 immunostaining (+) (weak immunoreactivity) (320 μm). (**B**): Photomicrograph of a cross-section of the hepatic tissue after administration of MoO_3_-NPs showed marked cytoplasmic hepatocyte staining of caspase-3, indicating marked apoptosis and toxicity in hepatocytes with sparing of the portal triad and inflammatory cells (++++) (very strong immunoreactivity) (320 μm). (**C**) Photomicrograph of a cross-section of the hepatic tissue after administration of CdCl_2_ showing strong (marked) cytoplasmic hepatocyte staining of caspase-3, indicating severe apoptosis resulting from toxicity in hepatocytes, without staining of nuclei, portal triad, or inflammatory cells (++++) (very strong immunoreactivity) (320 μm). (**D**) Photomicrograph of a cross-section of the hepatic tissues of RJ treated group showed negative hepatocyte staining of caspase-3 in hepatocytes, indicating the absence of toxicity (++++) (very strong immunoreactivity) (320 μm). (**E**) Photomicrograph of a cross-section of the hepatic tissues after administration of MoO_3_-NPs and RJ showed moderate cytoplasmic caspase-3 immunostaining (++) (moderate immunoreactivity) (320 μm). (**F**) Photomicrograph of a cross-section of the hepatic tissues after administration of a combination of CdCl_2_ and RJ showing moderate cytoplasmic caspase-3 immunostaining (++) (moderate immunoreactivity) (320 μm). (**G**) Photomicrograph of a cross-section of the hepatic tissues of the group treated with a combination of MoO_3_-NPs and CdCl_2_ with RJ showed mild to moderate caspase-3 immunostaining (+) (weak immunoreactivity) (DAB chromogen, Meyer’s hematoxylin counterstain) (320 μm).

The immunohistopathological examination of the liver tissue as shown in (Figure 11) for caspase-3 sections of rats showed marked positive staining for this marker in both the CdCl_2_ and/or MoO_3_-NPs treated groups. Meanwhile, negative staining in the control and RJ groups and mild staining in the combined treated groups of CdCl_2_ and/or MoO_3_-NPs and RJ.

**Figure 12:**
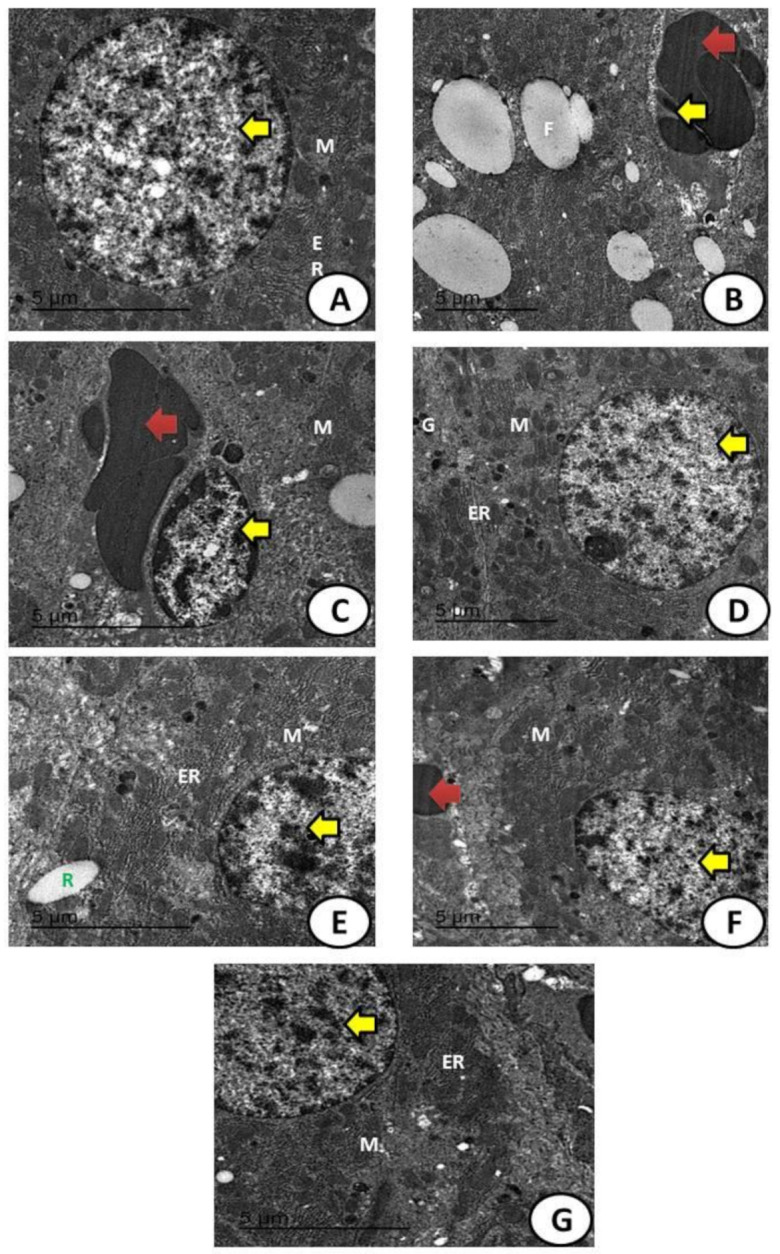
An electron micrograph of hepatic tissues of (**A**), the control group showed a normal nucleus (yellow arrow) with the continuous nuclear membrane and the appearance of normal-sized mitochondria (M) and endoplasmic reticulum (ER). (**B**) The MoO_3_-NPs treated group shows detached hepatic parenchyma with large fat droplets as a sort of fatty change (F) and the appearance of hemorrhage (red arrow) and necrotic nucleus (yellow arrow) with reduced mitochondria. (**C**) The CdCl_2_ treated group with apoptotic nuclei (yellow arrow) and hemorrhage (red arrow) as well as degenerated mitochondria (M). (**D**) The RJ treated group with a normal nucleus (yellow arrow) and continuous nuclear membrane, as well as normal-sized M, ER, and glycogen granules (g). (**E**) The MO-NPs and RJ treated groups demonstrated restoration of the normal-sized nucleus (yellow arrow) with mitochondria and ER, as well as partial MoO_3_-NP residues (R). (**F**) The CdCl_2_ and RJ treated groups show restoration of the normal-sized nucleus (yellow arrow) with the appearance of small red blood cells (red arrow). (**G**) The MoO_3_-NPs and CdCl_2_ followed by RJ treated group showed restoration of the normal-sized nucleus (yellow arrow) with the appearance of normal endoplasmic reticulum (ER) and mitochondria (M) with fewer fatty changes.

An electron micrograph of hepatic tissues of the control group showed a normal nucleus with the continuous nuclear membrane and the appearance of normal-sized mitochondria (M) and endoplasmic reticulum (ER) (Figure 12A). The MoO_3_-NPs treated group showed detached hepatic parenchyma with large fat droplets as a sort of fatty change, with residues of MoO_3_-NPs and the appearance of hemorrhage and necrotic nucleus with reduced M (Figure 12B). The CdCl_2_ treated group exhibited apoptotic nuclei and hemorrhage and degenerated M (Figure 12C). The RJ treated group had a normal nucleus and continuous nuclear membrane, as well as normal-sized M, ER, and glycogen granules (Figure 12D). The MoO_3_-NPs and RJ treated groups demonstrated restoration of the normal-sized nucleus with M and ER (Figure 12E). The CdCl_2_ and RJ treated groups showed restoration of the normal-sized nucleus with the appearance of small red blood cells (Figure 12F). The combination of MoO_3_-NPs and CdCl_2_ followed by the RJ treated group showed restoration of the normal-sized nucleus with the appearance of normal ER and M with fewer fatty changes (Figure 12G). A great improvement in the hepatic structure was observed in combined groups between MoO_3_-NPs and CdCl_2_ with RJ.

## 4. Discussion

Poisonous metals and environmental toxins are significantly associated with the incidence of hazardous diseases and a high risk of cancer. In spite of extensive uses of MoO_3_-NPs in a wide range of applications, limited studies are available on the toxic effects of MoO_3_-NPs, as well as their impact on the hepatic functions and oxidative stress. Cadmium is widely distributed in the blood and whole body, with excessive accumulation of free radicals and the generation of toxicity in different organs of the body, including the liver [33].

Several previous studies confirmed the concept of the beneficial and antioxidant effects of RJ [34,35]. Therefore, we aimed to evaluate the possible antioxidant and ameliorative effects of RJ against hepatic toxicity induced by either MoO_3_-NPs or/and CdCl_2_. Therefore, the present study was carried out to study the effects of combinations of CdCl_2_ and MoO_3_-NPS and their impact on hepatic function of histological structures and antioxidant enzyme levels. Our findings are of significant importance to alleviate the severe oxidative stress series, which is the cause of risky diseases due to exposure to environmental toxins and heavy metals.

The fundamental vibrational mode calculations of Mo=O, based on previous calculations of Segun et al. [36], and the existence of stretching and bending bonds was comparatively low since the samples were calcined at high temperatures. Schoonman [37] connected the dominion band at 868/cm with the vibration of Mo–O–Mo bridge bonds. According to Chithambararaj et al. [38], the absorption bands found at 1377 and 1633/cm are related to the vibration mode of the Mo–OH bond and the bending mode of adsorbed water, respectively.

The MoO_3_-NPs were dispersed in ethanol with a concentration of about 10^−4^ mol/L and then sonicated at room temperature for 30 min, and a colloidal solution was thus obtained; the blank was measured by using pure absolute ethanol. These data are in agreement with data presented in the literature by Bagheri et al. [39]. The grain size from SEM images is the domain formed by the agglomeration of nanocrystalline [40].

The data hepatic functions enzymes (AST, ALT, ALP, and LDH) indicated that cell membrane disruption occurred [33,41] after treatment with MoO_3_-NPs or CdCl_2_. The decrease in total protein might also be caused by inflammation of the hepatic tissues due to either MoO_3_-NPs or CdCl_2_ exposure, which greatly disturbs protein biosynthesis [42].

ALT is a specific enzyme marker of liver damage [43]. In the present study, the administration of either CdCl_2_ and/or MoO_3_-NPs led to a significant increment in ALT levels. Glucoprotein is a 57-KDa glycoprotein found in RJ; it plays an important role in stimulating liver regeneration and hepatocyte development. 

Previous studies demonstrated that RJ has a strong hepatoprotective effect against chemicals that lead to liver damage [44,45]. The hepatoprotective effects of RJ observed in this study are in complete agreement with previous results reported by Kanbur et al. [46], who confirmed that RJ and honey products declined the incidence of hepatic damage and oxidative CdCl_2_ and MoO_3_-Nps have toxic effects via the excessive production and generation of reactive oxygen species (ROS) through an imbalance in the antioxidant–oxidant status causing oxidation of biological biomolecules, as proteins, lipids, and DNA. CdCl_2_ combined MoO_3_-NPs exposure induces acute hepatotoxicity, apoptosis, necrosis, and hepatic infiltration, as shown in histological and histochemical sections [47,48].

Gaskill et al. [49] showed serious liver damage in cadmium treated groups, as evidenced by a considerable increase in the hepatic enzymes ALT, AST, and ALP when compared to the control group, which is consistent with our findings. As a result, it is subsequently determined that the hepatic enzyme impairment is due to higher oxidative stress indicators. This oxidative injury could be the main sign of hepatotoxicity induction in groups treated with MoO_3_-NPs or CdCl_2_. Lakshmi et al. [50] confirmed that exposure to CdCl_2_ elevated the liver biomarkers’ levels and declined the levels of total proteins. Hence, the study explains the liver injury caused by MoO_3_-NPs and/or CdCl_2_ treatments.

Our results also correlated well with the effects of MoO_3_-NPs that include the following changes: loss of mitochondrial potential and lysosomal membrane destabilization [51]. The histological changes in hepatic tissues exposed to MoO_3_-NPs also confirm the cytotoxic effects as observed by severe damage of liver tissue, severe loss of normal hepatic lobular architecture, and the appearance of large areas of hemorrhage, as well as necrosis in the hepatic tissues with an accumulation of bilirubin particles, indicating obstruction with the appearance of inflammatory cells.

The MRJP is a weak acidic glycoprotein that accounts for approximately 48% of RJ proteins [52]. Recently, the function of (MRJPs) of RJ, antimicrobial properties [53], medicinal value [54,55], and healthy aging and longevity [56] have been reported, and thus, RJ is considered a protective agent for heart vitality and adjusting lipid levels in the body. 

Dyslipidemia is a factor for heart disease that is aggravated by a poor diet. Atherosclerotic cardiovascular disease is caused by low HDL-c and high TG and LDL-c levels in the blood [57]. Many types of research have been conducted to see how RJ affects blood lipid concentrations, and they have discovered that RJ can lower TC levels while increasing HDL-c levels [58,59,60]. 

The MRJP’s suppression of TC absorption in the jejunum might be causing these alterations in the lipid profile. MRJP1 can also prevent bile acids from being reabsorbed [61]. Moreover, RJ could induce the upregulation of cholesterol 7-α-hydroxylase (CYP7A1) that could elevate the sensitivity of hepatic receptors for the synthesis of vLDL-c, precursors of LDL-C [62]. Since TC and LDL-c levels were lowered with intake treatment of RJ [63], RJ has been recommended as a hypocholesterolemic drug. Another study indicated that dietary RJ might reduce white adipose tissue and liver accumulation while increasing brown adipose tissue thermogenic capability in rats without changing their food or energy intake [64]. On the other hand, some previous studies found that the effects of RJ on lipid profiles are conflicting [62,63,64]. 

Antioxidants such as RJ are radical scavengers; they have the ability to inhibit lipid peroxidation and other free radicals that facilitate the progression of many diseases [63]. Previous studies showed that RJ proteins have antioxidant activity against lipid peroxidation via scavenging hydroxyl radicals [64], and they attributed this activity due to three dipeptides containing tyrosine residues. Additionally, Kanbur et al. [53] found that the antioxidant activity of RJ is not only due to its scavenging activity but also due to the effect of RJ on the inhibition of enzymes that elevate the peroxidation of endogenous lipids, as well as cytochrome P450 expression, which is one of the cellular sources of oxygen radicals. Thus, RJ products can be used to prevent and treat many liver dysfunctions since they are rich in natural antioxidant compounds.

The administration of CdCl_2_ to the blood resulted in a considerable increase in serum TNF-α. The current results concur with those of Alghasham et al. [65], who discovered that CdCl_2_ elevated TNF-, IL-6, and oxidative stress markers in rats. As a result, we believe that Cd poisoning increases the production of TNF- and IL-6, which are associated with oxidative stress in the body. RJ may have an anti-inflammatory effect by inhibiting the transcription of pro-inflammatory cytokines TNF- and IL-6 [66].

Free radicals trigger the development of many diseases and cause harmful effects that cause the peroxidation of biomembranes and DNA, which is the reason for tissue destruction. Antioxidants may prevent the body from several diseases caused by the destructive effects of free radicals [67]. Previous studies have reported that oxidative stress induced due to exposure to cadmium chloride cause different toxicities in different tissues [67]. Cd exposure induced cellular lesions and general aspects, including genotoxic effect [43] and oxidative damage [67]. Cadmium chloride that enters the body combines with metallothionein and then accumulates in soft tissue, such as the liver, which is the essential vital organ affected by exposure to a lot of poisons, as it is the organ responsible for detoxifying external toxic compounds [67].

The excessive generation of free radicals is considered as one of the mechanisms by which Cd induces toxicity [67]. Therefore, the co-exposure to CdCl_2_ and MoO_3_-NPs causes a more pronounced increase in hepatic oxidative stress. It shows that the increase in the free radical generation is due to the dual impact of CdCl_2_ and MoO_3_-NPs. Hepatica causes disturbances in the body’s metabolism, including an oxidant-antioxidant imbalance in the host to a higher degree.

Cadmium has been reported to cause adverse reactions at multiple cellular aspects and altered structure of biomolecules and their functions were induced by Cd via promoting the excessive generation of reactive oxygen species (ROS), thereby leading to oxidative stress [67]. MoO_3_-NPs administration confirmed the entrance of these nanoparticles into the liver tissues in agreement with Donald [68], who reported a high concentration of MoO_3_-NPs after circulation of the blood in the liver tissues. In the present study, Cd and MoO_3_-NPs caused histological alterations and disturbances in immune markers, and all these effects were reversed in the combined group treated with both CdCl_2_ and RJ, which confirms the hepatoprotective effect of RJ in alleviating hepatotoxicity and all these previous findings confirmed the structural alterations caused in the hepatic tissues intoxicated with MoO_3_-NPs and/or their combinations.

SOD is an antioxidant enzyme that has physiological activities, which include scavenging free radicals, fighting oxygen free radical damage to cells, as well as repairing damaged cells [69].

As an important antioxidant enzyme, CAT protects cells from toxic effects; the accumulation of H_2_O_2_ leads to hydroxyl radicals [70]. CAT can decompose hydrogen peroxide into molecular O_2_ and H_2_O, thereby protecting cells from hydrogen peroxide toxicity. Glutathione enzyme is an important intracellular antioxidant that protects cells from damage induced by ROS, peroxides, and toxins. GSH-Px is an important antioxidant enzyme, which catalyzes the reduction of H_2_O_2_ and organic H_2_O_2_ to H_2_O and alcohol to prevent oxidative damage of cells [71].

Oxidative stress has been recognized as one of the most important mechanisms of harm and toxicity generated by nanoparticle exposure and MDA is one of the final products of lipid peroxidation. The content of MDA reflects the degree of lipid peroxidation of cells in the body, which indirectly reflects the degree of cell damage [72]. The current results showed that MoO_3_-NPs exert toxicity in the hepatic tissues and these results agreed with the previous findings [73] and RJ exerted antioxidant effect against these hepatic alterations due to high antioxidant capacities of RJ as reported previously [74]. 

In this study, the results showed that MoO_3_-NPs and CdCl_2_ induced large amounts of reactive oxygen species production accompanied by a decrease of activities of antioxidant enzymes, including SOD, CAT and GPx, and an increase of lipid peroxidation marker (MDA). These findings illustrated the severe oxidative damage and cellular toxicity induced by a combination of both MoO_3_-NPs and CdCl_2_ and the ameliorative effects of RJ in the alleviation of hepatotoxicity and oxidative injury.

## 5. Conclusions

The obtained results clarified that both MoO_3_-NPs and CdCl_2_ induced hepatic toxicity with the excessive triggering of ROS that induced severe oxidative injury, histological alterations in the hepatic structure, and hepatic ultrastructure. These results are concurrent with obtaining normal biochemical levels in groups treated with either RJ or a combination of RJ and two xenobiotics (CdCl_2_ and MoO_3_-NPs). RJ is considered an essential potential source of natural antioxidants capable of frustrating the effects of oxidative injury, which is considered the main cause of many diseases.

## Figures and Tables

**Table 1:** Assessment of royal jelly (RJ) on serum enzyme activity of male rats treated with molybdenum nanoparticles (MoO_3_-NPs), cadmium chloride (CdCl_2_), or their combinations for 30 consecutive days.

Parameters	Control	MoO_3_-NPs	CdCl_2_	RJ	MoO_3_-NPs + RJ	CdCl_2_ + RJ	MoO_3_-NPs + CdCl_2_+ RJ
ALT (U/L)	12.12 ± 0.07 ^f^	156.88 ± 2.52 ^b^	184.30 ± 2.05 ^a^	13.25 ± 0.14 ^f^	29.28 ± 0.92 ^d^	35.39 ± 1.72 ^c^	23.64 ± 0.92 ^e^
AST (U/L)	12.45 ± 0.16 ^f^	282.22 ± 1.93 ^a^	273.40 ± 3.86 ^b^	12.45 ± 0.12 ^f^	34.17 ± 2.78 ^d^	37.12 ± 2.52 ^c^	27.30 ± 1.83 ^e^
ALP (U/L)	26.87 ± 0.44 ^f^	194.85 ± 2.32 ^a^	179.92 ± 3.07 ^b^	23.17 ± 1.40 ^g^	27.36 ± 0.58 ^ef^	37.23 ± 1.44 ^c^	30.11 ± 0.877 ^d^
LDH (U/L)	110.04 ± 4.29 ^d^	525.60 ± 18.66 ^b^	604.47 ± 36.14 ^a^	102.43 ± 1.51 ^d^	193.99 ± 7.96 ^c^	213.06 ± 13.12 ^c^	141.56 ± 14.311 ^d^
Total proteins (g/dL)	8.05 ± 0.15 ^a^	4.25 ± 0.28 ^c^	4.36 ± 0.41 ^c^	7.67 ± 0.20 ^a^	5.61 ± 0.34 ^b^	5.67 ± 0.26 ^b^	7.06 ± 0.57 ^a^

Values are expressed as mean ± SE; *n* = 10 for each treatment group. ALT, alanine aminotransferase; AST, aspartate aminotransferase; ALP, alkaline phosphatase; and LDH, lactate dehydrogenase. Symbols of different letters are indicated to be significantly different compared to the control group and other treated groups (*p* ≤ 0.05).

**Table 2:** The effect of Royal Jelly (RJ) on the serum lipid profile of male rats given molybdenum nanoparticles (MoO_3_-NPs), cadmium chloride (CdCl_2_), or a combination of the two for 30 consecutive days.

Parameters (mg/dL)	Control	MoO_3_-NPs	CdCl_2_	RJ	MoO_3_-NPs + RJ	CdCl_2_ + RJ	MoO_3_-NPs + CdCl_2_ + RJ
TG	76.01 ± 1.66 ^c^	185.48 ± 5.72 ^a^	171.11 ± 12.66 ^a^	72.07 ± 2.04 ^c^	147.09 ± 5.57 ^b^	139.34 ± 4.47 ^b^	133.99 ± 10.23 ^b^
TC	134.19 ± 4.40 ^d^	270.91 ± 2.13 ^a^	266.76 ± 8.99 ^b^	132.96 ± 5.25 ^d^	173.26 ± 2.19 ^c^	196.24 ± 9.05 ^b^	146.86 ± 6.12 ^d^
HDL-c	39.30 ± 0.60 ^a^	26.30 ± 1.54 ^e^	23.18 ± 0.866 ^f^	36.88 ± 1.47 ^bc^	29.02 ± 1.17 ^de^	30.09 ± 1.02 ^d^	34.61 ± 0.799 ^c^
LDL-c	28.91 ± 1.02 ^d^	40.74 ± 1.28 ^a^	38.94 ± 1.84 ^b^	26.09 ± 0.96 ^d^	34.41 ± 0.82 ^c^	34.16 ± 1.20 ^c^	27.95 ± 1.01 ^d^
vLDL-c	15.41 ± 0.211 ^f^	35.79 ± 0.688 ^b^	38.34 ± 0.60 ^a^	14.33 ± 0.44 ^f^	28.93 ± 0.43 ^c^	26.81 ± 0.76 ^d^	22.14 ± 0.47 ^e^

Values are expressed as mean ± SE; *n* = 10 for each treatment group. HDL-c; High-density lipoprotein, LDL-c; Low-density lipoprotein, and vLDL-c; very-low-density lipoprotein. Symbols are different alphabetically to indicate a significant comparison compared to the control group and other treated groups (*p* < 0.05).

**Table 3:** The effects of Royal Jelly (RJ) on serum pro-inflammatory levels in male rats treated with molybdenum nanoparticles (MoO_3_-NPs), cadmium chloride (CdCl_2_), or combinations of the two for 30 consecutive days.

Groups	IL-6 (Pg/g)	TNF-α (Pg/g)	CRP (mg/L)
Control	3.30 ± 0.13 ^d^	5.28 ± 0.10 ^e^	4.24 ± 0.11 ^e^
MoO_3_-NPs	27.40 ± 0.62 ^a^	32.15 ± 0.54 ^b^	26.40 ± 0.99 ^b^
CdCl_2_	22.98 ± 0.59 ^b^	36.79 ± 0.69 ^a^	28.81 ± 0.49 ^a^
RJ	3.02 ± 0.03 ^d^	5.74 ± 0.28 ^e^	4.74 ± 0.27 ^e^
MoO_3_-NPs + RJ	8.06 ± 0.15 ^c^	19.92 ± 0.53 ^c^	17.30 ± 0.46 ^c^
CdCl_2_ + RJ	7.80 ± 0.28 ^c^	19.44 ± 1.18 ^c^	16.04 ± 0.499 ^c^
MoO_3_-NPs + CdCl_2_ + RJ	7.33 ± 0.53 ^c^	15.85 ± 0.399 ^d^	13.81 ± 0.91 ^d^

Values are expressed as mean ± SE; *n* = 10 for each treatment group. TNF-α; Tumor necrosis factor Alpha, IL-6; interleukin-6, and CRP; C-Reactive protein. Different alphabetical symbols indicate significant differences compared to the control group and other treated groups (*p* < 0.05).

**Table 4:** The effect of Royal Jelly (RJ) on the oxidant and antioxidant status of male rats’ livers after treatment with molybdenum nanoparticles (MoO_3_-NPs), cadmium chloride (CdCl_2_), and combinations of the two for 30 consecutive days.

Groups	MDA (nmoles of MDA/g)	CAT (nmol/g of Protein/min)	SOD (U/g of Protein)	GPx (nmol/g of Protein/min)
Control	5.53 ± 0.22 ^e^	8.19 ± 0.27 ^a^	19.69 ± 0.62 ^b^	14.13 ± 0.69 ^a^
MoO_3_-NPs	33.12 ± 1.09 ^b^	3.09 ± 0.66 ^d^	7.57 ± 0.44 ^e^	6.52 ± 0.58 ^d^
CdCl_2_	38.83 ± 0.65 ^a^	3.35 ± 0.44 ^d^	6.40 ± 0.39 ^e^	5.36 ± 0.98 ^d^
RJ	5.10 ± 0.065 ^e^	8.17 ± 0.21 ^a^	21.76 ± 0.65 ^a^	14.25 ± 1.69 ^a^
MoO_3_-NPs +RJ	18.24 ± 0.49 ^c^	5.02 ± 0.55 ^c^	11.41 ± 0.50 ^d^	8.28 ± 1.58 ^c^
CdCl_2_ + RJ	20.13 ± 0.68 ^c^	5.85 ± 0.48 ^c^	11.27 ± 0.89 ^d^	9.25 ± 1.65 ^c^
MoO_3_-NPs + CdCl_2_ + RJ	16.03 ± 0.96 ^d^	7.25 ± 0.85 ^bc^	16.70 ± 0.58 ^c^	12.65 ± 0.98 ^b^

Values are expressed as mean ± SE; *n* = 10 for each treatment group. SOD; superoxide dismutase, MDA; malondialdehyde, GPx; glutathione peroxidase, CAT; catalase. Different alphabetical symbols indicate significant differences compared to the control group and other treated groups (*p* < 0.05).

## Data Availability

Data are available upon request from the corresponding author.
